# Second-order morphometric similarity networks predict response to transcutaneous auricular vagus nerve stimulation in major depressive disorder: a two-center study

**DOI:** 10.3389/fpsyt.2026.1858627

**Published:** 2026-07-08

**Authors:** Chunchen Liu, Yu Xiong, Tianjiao Xu, Jifei Sun, Yue Ma, Jun Liu, Weihui Li, Yaxuan Xu, Meng Zhao, Jiudong Cao, Yukang Zhang, Lei Zhang, Jiazheng Li, Xiaoling Wang, Xin Wang, Kai Sun, Changbin Yu, Jiliang Fang

**Affiliations:** 1 College of Medical Information and Artificial Intelligence, Jinan Central Hospital Affiliated to Shandong First Medical University, Shandong First Medical University and Shandong Academy of Medical Sciences, Jinan, China; 2 School of Clinical Medicine, Chengdu University of Traditional Chinese Medicine, Chengdu, China; 3 Guang’anmen Hospital, China Academy of Chinese Medical Sciences, Beijing, China; 4 Shunyi Hospital, Beijing Hospital of Traditional Chinese Medicine, Beijing, China; 5 Beijing Children’s Hospital, Capital Medical University, National Center for Children’s Health, Beijing, China; 6 National Clinical Research Center for Mental Disorders, The Second Xiangya Hospital of Central South University, Changsha, China; 7 School of Ophthalmology, Shandong First Medical University and Shandong Academy of Medical Sciences, Jinan, China; 8 Institute of Medical Genomics, Biomedical Sciences College and Shandong Medicinal Biotechnology Centre, First Affiliated Hospital of Shandong First Medical University, Shandong First Medical University and Shandong Academy of Medical Sciences, Jinan, China; 9 Shandong Engineering Research Center of Intelligent Surgery, The First Affiliated Hospital of Shandong First Medical University, Jinan, China; 10 Shandong Data Open Innovative Application Laboratory, The First Affiliated Hospital of Shandong First Medical University, Jinan, China; 11 Shandong Key Laboratory of Digital Diagnosis and Treatment of Thoracic Oncology, The First Affiliated Hospital of Shandong First Medical University, Jinan, China; 12 China Foundation for Youth Entrepreneurship and Employment-Incaier Public Welfare Fund, Beijing, China

**Keywords:** major depressive disorder, morphometric similarity network, orbitofrontal cortex, transcutaneous auricular vagus nerve stimulation, treatment response prediction

## Abstract

**Introduction:**

Transcutaneous auricular vagus nerve stimulation (taVNS) is a promising neuromodulation therapy for major depressive disorder (MDD), but reliable predictors of treatment response are lacking. Second-order morphometric similarity networks (MSN-II), which capture higher-order structural covariance patterns, may provide novel predictive biomarkers.

**Methods:**

A total of 122 antidepressant-free MDD patients from two centers (Site A, n = 92; Site B, n = 30) underwent structural MRI before and after 8 weeks of taVNS. Baseline MSN-II nodal strength within 26 limbic regions was extracted from T1-weighted images. A LASSO logistic regression model was trained in Site A and externally validated in Site B. Treatment response was defined as ≥60% HAMD-17 reduction.

**Results:**

MSN-II showed significant training performance (AUC = 0.792 ± 0.158; permutation P < 0.001) and generalized to external validation (AUC = 0.856, 95% CI: 0.693–0.978). MSN-I showed comparable performance (external AUC = 0.804). MSN-II also showed higher external validation performance than ReHo, ALFF, subcortical volumes, and baseline HAMD-17. The left orbitofrontal cortex area 13 (L_OFC_A13; β = –0.649) was the strongest predictor among four retained features. Non-responders showed higher baseline MSN-II in L_OFC_A13 (P = 0.021) and significant post-treatment decreases (P < 0.001), whereas responders remained stable (Time × Group interaction, P = 0.005).

**Discussion:**

MSN-II features from limbic regions provide promising cross-site prediction of taVNS response in MDD, with L_OFC_A13 emerging as a key biomarker. These findings support the potential of MSN-based approaches for individualized neuromodulation treatment planning.

## 1 Introduction

Depressive disorders pose a major global public health burden and are among the leading contributors to disability worldwide (1). Although pharmacological and psychotherapeutic treatments have advanced considerably, a substantial proportion of patients still fail to respond adequately to first-line interventions, with recent reviews estimating inadequate response rates of approximately 30%–50% (2). This gap has motivated interest in non-invasive neuromodulation as a complement to existing therapies. Among these, transcutaneous auricular vagus nerve stimulation (taVNS) has emerged as a promising option for MDD, with clinical studies reporting antidepressant benefits and favorable safety profiles (3, 4). Yet response varies widely across patients, highlighting the need for biomarkers that can guide patient selection before treatment (5).

Neuroimaging-based prediction of treatment response has been explored using structural and functional measures, including gray matter volume, functional connectivity, and regional homogeneity (6–8). Beyond conventional MRI, emerging neuroimaging modalities have also demonstrated diagnostic potential for brain diseases (9), highlighting the broader landscape of neuroimaging biomarker development. Morphometric similarity networks (MSN) provide a framework for characterizing structural brain organization by quantifying interregional similarity across multiple morphometric properties derived from T1-weighted MRI (10). Building on previous multi-modal MRI analysis for psychiatric classification (11), recent work from our group introduced second-order MSN (MSN-II), which captures higher-order structural organization by integrating multi-texture connectivity profiles across brain regions, and demonstrated its potential for mood disorder differentiation (12). However, the potential of MSN-II for predicting taVNS response in MDD remains unclear.

Beyond methodological considerations, the selection of brain regions is also important for treatment response prediction. Previous neuroimaging studies have implicated several cortico-limbic regions in antidepressant outcomes, including the anterior cingulate cortex, hippocampus, amygdala, and prefrontal cortex (13, 14). Among these regions, the orbitofrontal cortex (OFC) has attracted particular attention because of its dense reciprocal connections with limbic structures and its role in reward valuation, emotion regulation, and decision-making, which are frequently disrupted in MDD (15–17). However, whether the network-level organization of the OFC and related limbic regions can predict response to taVNS has not been investigated.

This study aimed to develop and externally validate an MSN-based prediction model for taVNS response in MDD, with a focus on second-order MSN (MSN-II) features derived from limbic regions. We compared the predictive performance of MSN-II against MSN-I, conventional structural and functional MRI measures, baseline clinical severity and examined longitudinal MSN-II changes and brain-symptom associations related to treatment response.

## 2 Materials and methods

### 2.1 Participants

Patients with MDD were recruited from outpatient departments of Guang’anmen Hospital of China Academy of Chinese Medical Sciences (Site A) and Xiangya Second Hospital of Central South University (Site B) between January 2018 and December 2024. All patients met DSM-5 diagnostic criteria for MDD, confirmed using the Structured Clinical Interview for DSM-5 (SCID-5).

Inclusion criteria were: (1) age 18 to 70 years; (2) baseline HAMD-17 score greater than 7; and (3) antidepressant-free at baseline (no antidepressant medication within 1 month before taVNS initiation), including treatment-naive first-episode MDD or prior MDD with antidepressant-related remission. Exclusion criteria included contraindications to MRI, pregnancy, major neurological disorders, substance abuse or dependence, and severe medical illness.

Among the 172 initially enrolled participants, 122 were included in the final analysis because they completed the full 8-week taVNS treatment protocol and had complete pre- and post-treatment MRI and clinical assessments. The remaining 50 participants were not included because complete evaluable longitudinal imaging and/or clinical data were unavailable. Available records suggested that the main reasons included incomplete MRI acquisition, withdrawal or loss to follow-up, MRI quality issues, and incomplete follow-up clinical assessments. Because detailed category-specific counts could not be reliably reconstructed from the retrospective records, we did not report unsupported numerical breakdowns. Finally, these 122 participants comprised 92 patients from Site A (training cohort) and 30 patients from Site B (external validation cohort).

**Figure 1:**
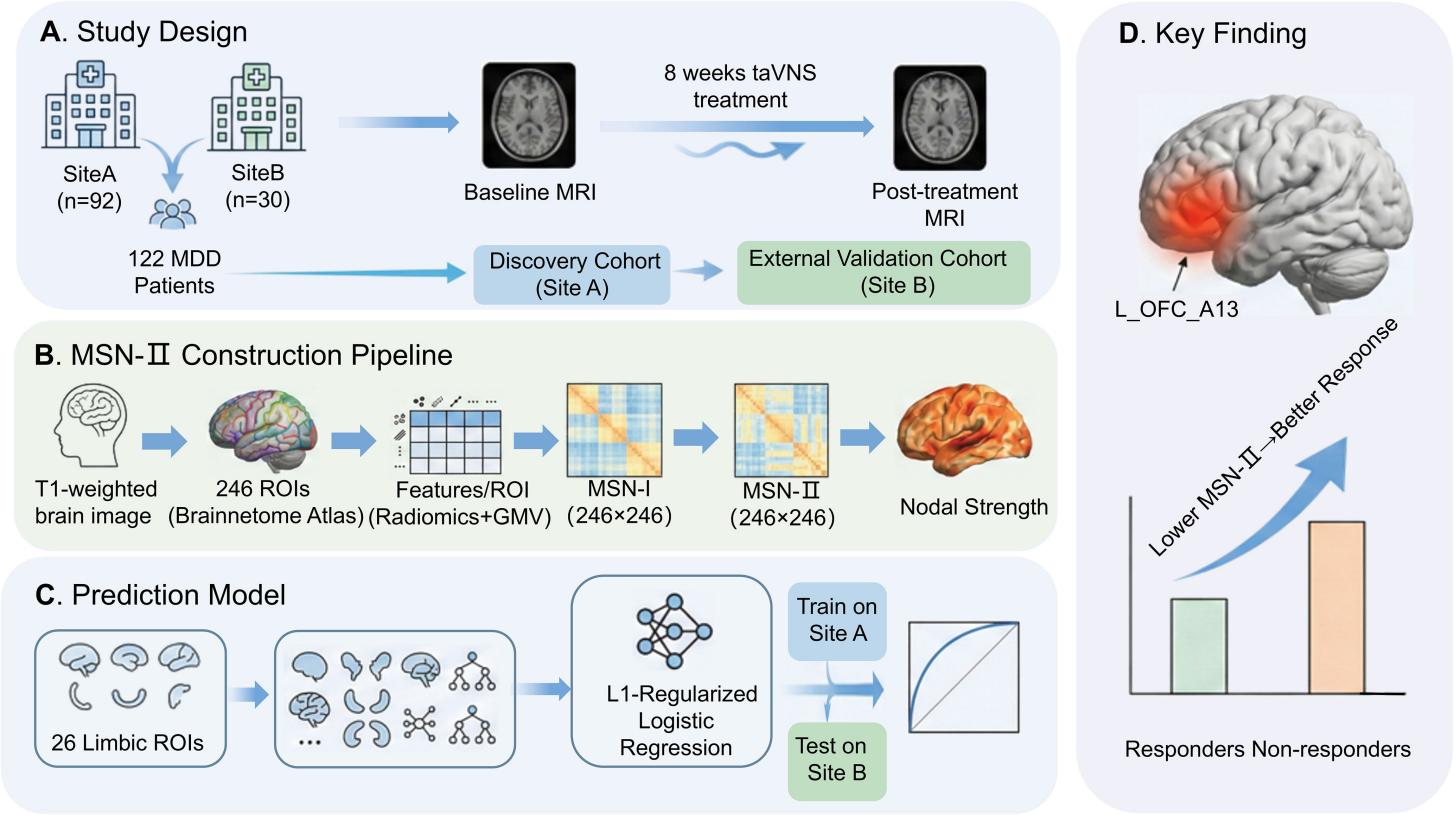
Study workflow. Overview of the study design, including participant recruitment, taVNS treatment, MRI acquisition, feature extraction, model training in Site A, and external validation in Site B. **(A)** Study design, **(B)** MSN-II construction pipeline, **(C)** Prediction model, and **(D)** Key finding.

The study was approved by the Ethics Committee of Guang’anmen Hospital (Approval No.: 2017-021-SQ) and registered with the China Clinical Trial Registry (ChiCTR1800014277). All participants provided written informed consent in accordance with the Declaration of Helsinki. The overall study workflow is shown in **Figure 1**.

### 2.2 taVNS treatment protocol

Patients received bilateral taVNS targeting the cymba conchae using an SDZ-IIB electronic acupuncture device (Suzhou Huato Medical Equipment Co., Ltd., China). Stimulation was applied at alternating dense–disperse frequencies of 4/20 Hz. The current intensity (4–6 mA) was adjusted according to individual tolerance, with a pulse width below 1 ms. Each session lasted 30 minutes and was performed twice daily for 8 weeks.

### 2.3 Clinical assessment and outcome definition

Clinical assessments were performed at baseline (pre-treatment) and after 8 weeks of taVNS (post-treatment). The primary outcome measure was the 17-item Hamilton Depression Rating Scale (HAMD-17). Additional assessments included the Hamilton Anxiety Rating Scale (HAMA), Self-rating Depression Scale (SDS), and Self-rating Anxiety Scale (SAS).

Treatment response was defined as at least a 60% reduction in HAMD-17 scores from baseline to week 8. This stringent threshold, more conservative than the commonly used 50% criterion (18), was adopted to reduce label noise and phenotypic heterogeneity in predictive modeling (19–21) (Equation 1):

(1) $$\begin{array}{c} Reduction\;rate\;(\%)=\frac{HAMD_{pre}-HAMD_{post}}{HAMD_{pre}}\times 100\% \end{array}$$

For brain-symptom association analyses, absolute HAMD-17 reduction (ΔHAMD-17 = HAMD_pre_ − HAMD_post_) was used as the continuous outcome measure.

### 2.4 MRI acquisition

Structural MRI data were acquired at both sites using 3T Siemens Magnetom Skyra scanners with harmonized protocols. High-resolution 3D T1-weighted images were obtained using a magnetization-prepared rapid gradient echo (MPRAGE) sequence with the following parameters: TR = 2500 ms; TE = 2.98 ms; flip angle = 7 degrees; FOV = 256 × 256 mm²; matrix size = 256 × 256; slice thickness = 1 mm; 192 sagittal slices; acquisition time approximately 6 minutes. Resting-state functional MRI data were also acquired at both sites using an echo-planar imaging (EPI) sequence with the following parameters: TR = 2000 ms; TE = 30 ms; flip angle = 90°; FOV = 240 × 240 mm²; matrix size = 64 × 64; slice thickness = 3 mm; slice gap = 1 mm; 32 axial slices; 200 volumes; acquisition time approximately 6 minutes and 46 seconds. Participants wore noise-canceling headphones and were instructed to remain still with eyes closed and stay awake during scanning.

### 2.5 Structural preprocessing and MSN-II feature extraction

Structural MRI preprocessing was performed using a stepwise pipeline. First, FastSurfer v2.4.2 (deepmi/fastsurfer Docker image) (22) was used for deep learning-based cortical reconstruction and brain tissue segmentation with FreeSurfer-compatible outputs. Second, fMRIPrep v25.0.0 (Docker image) (23) was used to generate preprocessed T1-weighted images, gray matter probability maps, and spatial normalization transforms (ANTs, as implemented in fMRIPrep). Third, the Brainnetome Atlas v1.0 (24) in MNI space was transformed to each participant’s native T1 space using inverse ANTs transforms (antsApplyTransforms), yielding subject-specific native-space ROI masks. Fourth, these subject-specific ROI masks were used for ROI-wise radiomics feature extraction and subsequent MSN construction.

We selected 26 cortical ROIs assigned to the limbic network (Network 5) in the Yeo 7-network parcellation (25) as mapped onto the Brainnetome Atlas (24). These regions encompass orbitofrontal, lateral frontopolar, temporal pole, inferior temporal, fusiform, and parahippocampal areas. The complete list of the 26 limbic ROIs, including Brainnetome label IDs, abbreviations, and anatomical descriptions, is provided in **Supplementary Table 1**. Because the Yeo 7-network assignment is hemisphere-specific, three regions (A10l, A11l, A35/36c) were represented only in the left hemisphere and one region (A20il) only in the right hemisphere, yielding an asymmetric set of 14 left-hemisphere and 12 right-hemisphere ROIs.

Radiomics features were extracted using PyRadiomics v2.2.0 (26). Extraction settings included resampling to 1 mm isotropic resolution (B-spline interpolation), a gray-level bin width of 5, intensity normalization (scale = 100), and mask correction with a minimum ROI size of 5 voxels. Five texture feature groups were computed for each ROI: gray-level co-occurrence matrix (GLCM; n = 24), gray-level run-length matrix (GLRLM; n = 16), gray-level size-zone matrix (GLSZM; n = 16), gray-level dependence matrix (GLDM; n = 14), and neighborhood gray-tone difference matrix (NGTDM; n = 5), yielding 75 spatial texture features per ROI. First-order intensity statistics were excluded from MSN construction because they reflect marginal intensity distributions rather than spatial texture patterns. ROI-wise gray matter volume (GMV) was extracted separately from tissue segmentation maps and retained for comparison analyses. Within each texture group, features were z-scored across ROIs within each subject, and quality control confirmed consistent ROI counts and identical feature columns across participants.

Two types of morphometric similarity networks were constructed to evaluate their respective predictive performance. For MSN-I (first-order morphometric similarity network), a 246×246 symmetric matrix was constructed for each subject, where each entry represented the Pearson correlation between the 76-dimensional morphometric feature vectors (75 radiomics features + GMV) of the corresponding ROI pair (Equation 2):

(2) $$\begin{array}{c} S^{\left(I\right)}(i,j)=Pearson(x_{i},x_{j}),\;i,j\in \end{array}\{1,\ldots ,246\}$$

where $x_{i}$ denotes the z-scored 76-dimensional feature vector of ROI i.

For MSN-II (second-order morphometric similarity network), we employed a hierarchical multi-texture integration approach. First, for each of the five texture groups (GLCM, GLDM, GLRLM, GLSZM, NGTDM), an intermediate similarity matrix was computed by correlating the group-specific feature vectors across ROIs. For each texture group g, the off-diagonal row vector f(i,g) (length = 245) was extracted from ROI i and standardized to zero mean and unit variance. The optimal combination of texture groups (GLCM+GLDM+GLRLM) was selected by evaluating all combinations of 3–5 groups (16 combinations total) via 10-fold cross-validation within the Site A training cohort, maximizing mean AUC. The standardized off-diagonal vectors from the three selected texture groups were concatenated to form a composite profile vector h(i) of length 735 for each ROI. The MSN-II matrix was computed by calculating the Pearson correlation between composite profile vectors of each pair of regions (Equation 3).

(3) $$\begin{array}{c} S^{\left(II\right)}(i,j)=Pearson(h_{i},h_{j}) \end{array}$$

For both MSN-I and MSN-II, nodal strength for ROI i was defined as the mean off-diagonal value (Equation 4):

(4) $$\begin{array}{c} S_{i}=\frac{1}{N-1}\sum_{j=1,\;j\neq i}^{N}S(i,j),\;\;\;\;N=246 \end{array}$$

**Figure 2:**
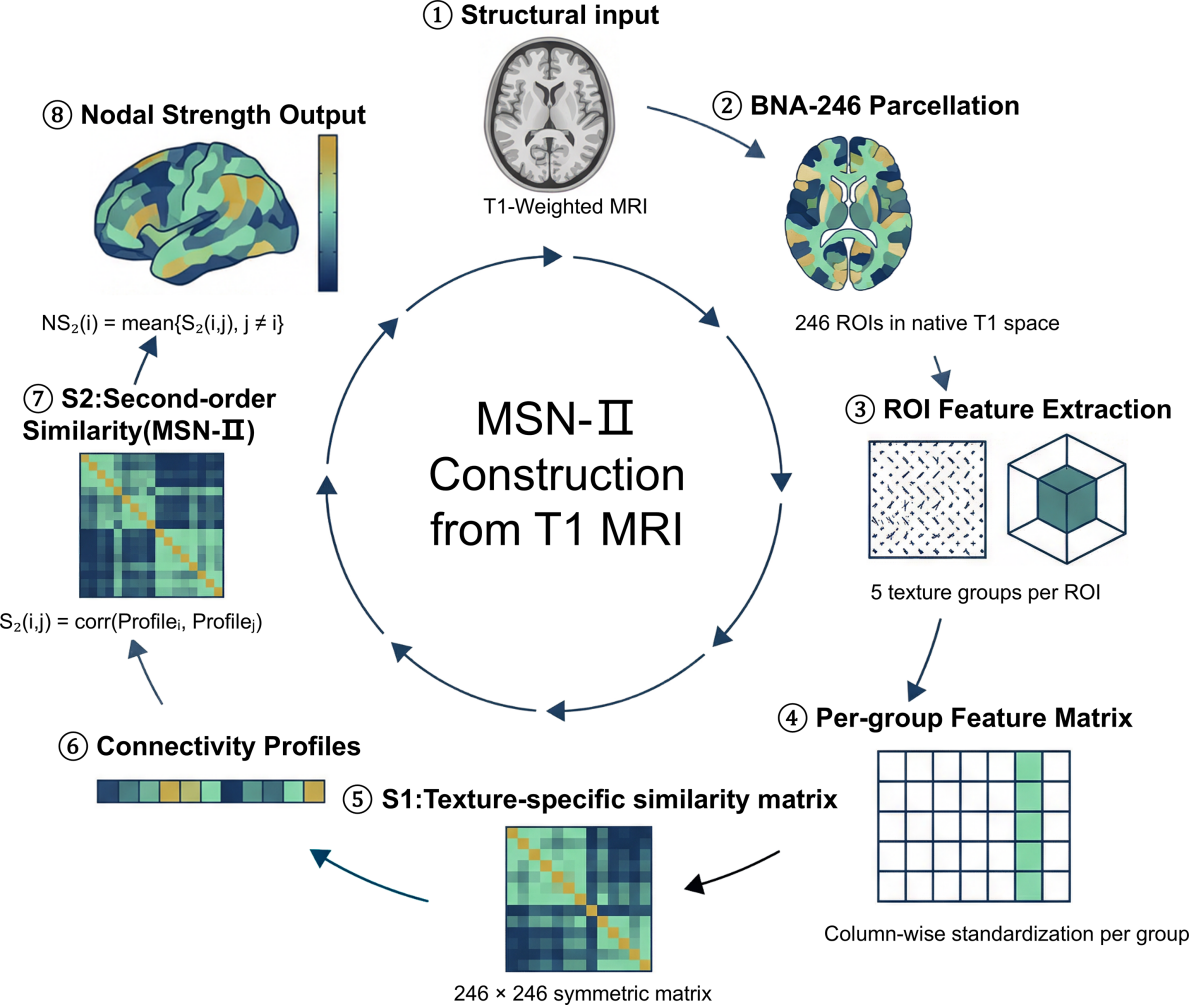
MSN-II feature extraction pipeline. MSN-II feature extraction pipeline. T1-weighted images were parcellated into 246 Brainnetome Atlas regions. Five radiomics texture groups (GLCM, GLDM, GLRLM, GLSZM, NGTDM) were extracted per ROI using PyRadiomics. For each texture group, a first-order MSN (MSN-I) was constructed via inter-ROI Pearson correlations. MSN-II was derived by concatenating row-standardized off-diagonal connectivity profiles from the selected texture groups (GLCM + GLDM + GLRLM, chosen via 10-fold inner cross-validation) and computing inter-ROI Pearson correlations on the concatenated profiles. MSN-II nodal strength was defined as the mean off-diagonal value per ROI. The optimal texture combination was selected exclusively within the Site A training cohort.

For comparison analyses, conventional morphometric features were also extracted from FastSurfer outputs. Subcortical volumetric features included bilateral hippocampus, amygdala, and thalamus volumes derived from the ASEG segmentation (6 features total). Resting-state functional MRI features were additionally extracted for comparison. Regional homogeneity (27) and amplitude of low-frequency fluctuations (28) were computed for each of the 246 Brainnetome Atlas ROIs using fMRIPrep-preprocessed resting-state data. For subjects with multiple runs, values were averaged across runs. ReHo and ALFF values from all 246 Brainnetome Atlas ROIs were used as input features for prediction modeling; no limbic ROI selection was applied for these functional measures, given the whole-brain nature of resting-state signal. The overall structural preprocessing and MSN-II feature extraction workflow is illustrated in **Figure 2**.

### 2.6 Prediction modeling and statistical analysis

An L1-regularized (LASSO) logistic regression model was used for binary classification of treatment response. To mitigate the moderate class imbalance between responders and non-responders in the training cohort, class-specific loss weights were assigned inversely proportional to their respective sample frequency. The regularization parameter C was independently optimized for each feature set via inner 5-fold cross-validation within the Site A training cohort, with C selected from {0.001, 0.005, 0.01, 0.05, 0.1, 0.3, 0.5, 0.7, 1.0, 2.0, 5.0} to maximize mean AUC. This per-feature optimization ensures that the regularization strength is appropriate for the dimensionality and signal characteristics of each feature set. After selecting the optimal C, model generalizability was assessed using 10-fold cross-validation with the fixed hyperparameter within the training cohort. To assess statistical significance, permutation testing (2,000 permutations) was performed for each feature set using the previously selected optimal C, in which class labels were randomly shuffled and 10-fold cross-validation repeated with the fixed hyperparameter to generate a null distribution of AUC scores (29). Feature sets with permutation P < 0.05 were considered to have predictive power significantly above chance. For MSN-II, the regularization parameter was fixed at C = 0.3, which was used both during texture group combination selection and final model training, ensuring consistency between the feature engineering and modeling stages.

To evaluate the predictive value of MSN-II, we compared the performance of the model built with MSN-II against the models built with MSN-I nodal strength, ReHo, ALFF, subcortical volumes (6 FreeSurfer-derived features), baseline HAMD-17, ROI-wise GMV, and cortical morphometry (5 FreeSurfer-derived features including mean thickness, hemisphere-wise curvature and sulcal depth). MSN-I and MSN-II nodal strength were evaluated within the 26 limbic ROIs. ReHo and ALFF were analyzed across all 246 Brainnetome Atlas ROIs. GMV, subcortical volumes, and cortical morphometry were subject-level measures derived from FreeSurfer segmentation. All features were evaluated using identical modeling pipelines. Model performance was evaluated using the area under the receiver operating characteristic curve (AUC), accuracy, sensitivity, specificity, positive predictive value (PPV), and negative predictive value (NPV). For demographic and clinical characterization, variables were compared between Site A and Site B using independent-samples T-tests for continuous variables and chi-square tests for categorical variables. To examine baseline neuroimaging differences, MSN-II nodal strength values were compared between responders and non-responders using independent-samples T-tests, with Cohen’s d reported as a measure of effect size. Treatment-related changes in MSN-II from baseline to week 8 were assessed using paired-samples T-tests within each group (responders and non-responders, separately). The between-group difference in the magnitude of MSN-II change (ΔMSN-II = pre-treatment − post-treatment) was evaluated using independent-samples T-tests. Brain–symptom coupling was assessed using a multiple linear regression model in the full sample, with ΔHAMD-17 as the outcome variable and ΔMSN-II, response group (binary: 1 = responder, 0 = non-responder), and their interaction term as predictors. A significant interaction term indicates that the brain–symptom association differs between groups. Within-group Pearson correlations between ΔMSN-II and ΔHAMD-17 were additionally computed for descriptive purposes. Feature importance in the prediction model was quantified using absolute LASSO regression coefficients and SHAP values. External AUC comparisons were performed using paired DeLong tests when predictions were available for the same external participants, and using an AUC comparison test when appropriate. All prediction modeling was implemented in scikit-learn v1.6.1, and model interpretation used SHAP v0.48.0.

## 3 Results

### 3.1 Demographic and clinical characteristics

Demographic and clinical characteristics are summarized in **Table 1**. There was no significant difference in age, sex distribution, education, baseline HAMD-17, HAMA, or SAS scores between Site A (training cohort, n = 92) and Site B (external validation cohort, n = 30). Baseline SDS scores were slightly higher in Site A (54.2 ± 8.3 vs. 51.0 ± 5.2, P = 0.047). Given that SDS is a self-report scale, this small difference may partly reflect subjective reporting variability, whereas clinician-rated HAMD-17 and HAMA scores did not differ significantly between sites. Treatment response rates were comparable between sites, supporting overall cohort comparability for external validation.

**Table 1:** Demographic and clinical characteristics.

Variable	Site A (n=92)	Site B (n=30)	t/χ²	P
Age (years)	35.7 ± 13.5	31.2 ± 9.0	1.71	0.090
Sex, male/female, n (%)	28 (30.4)/64 (69.6)	14 (46.7)/16 (53.3)	1.97	0.160
Education (years)	14.5 ± 2.3	14.1 ± 3.2	0.68	0.501
HAMD-17 baseline	21.2 ± 3.8	20.2 ± 4.1	1.19	0.237
HAMA baseline	20.2 ± 6.8	21.0 ± 7.8	-0.50	0.618
SDS baseline	54.2 ± 8.3	51.0 ± 5.2	2.01	0.047
SAS baseline	51.2 ± 7.0	49.0 ± 5.2	1.63	0.105
Response rate (60%)	54/92 (58.7%)	19/30 (63.3%)	0.06	0.814

Response defined as ≥60% HAMD-17 reduction.

### 3.2 Prediction performance and feature importance

**Figure 3:**
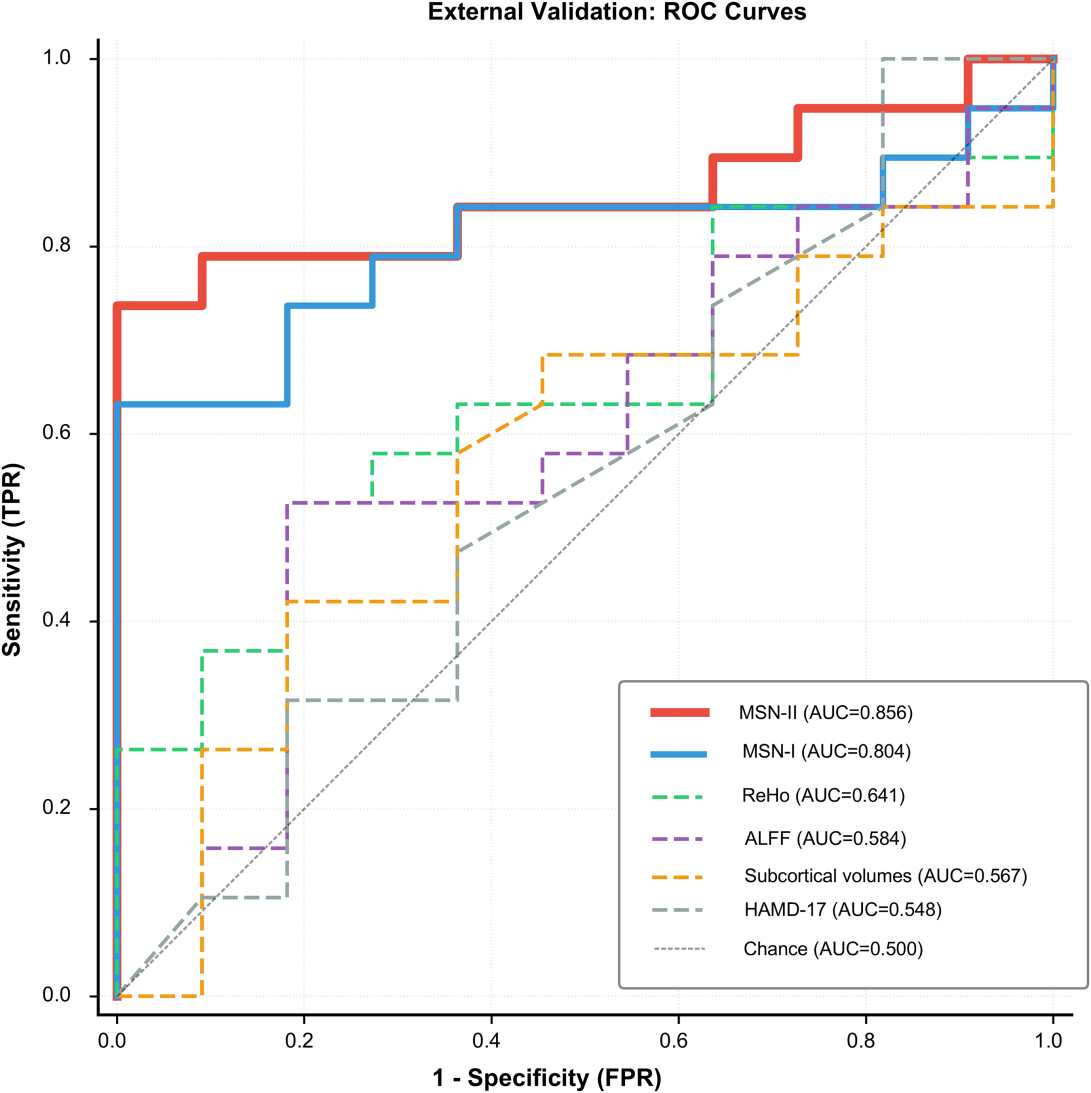
Receiver operating characteristic (ROC) curves for external validation. External validation ROC curves for treatment-response prediction (validation cohort, n = 30). MSN-II achieved the highest numerical AUC (0.856), followed by MSN-I (0.804). Subcortical volumes refer to six FreeSurfer-derived whole-structure subcortical volume measures, including bilateral hippocampus, amygdala, and thalamus, and are distinct from Brainnetome ROI-wise GMV. The ROC curves represent benchmark comparisons across candidate predictor families rather than dimension-matched anatomical comparisons. Asterisks indicate significant permutation test (P < 0.05) in the training cohort. Dashed diagonal = chance level.

External validation results are summarized in **Table 2** and **Figure 3**. Among all feature sets evaluated, MSN-II and MSN-I from limbic regions demonstrated significant predictive performance, while conventional measures did not. In 10-fold cross-validation within the Site A training cohort, MSN-II achieved a mean AUC of 0.792 (SD = 0.158; permutation P < 0.001). In the independent external validation cohort, the MSN-II model achieved an AUC of 0.856 (95% CI: 0.693–0.978), with an accuracy of 83.3%, sensitivity of 73.7%, and specificity of 100.0%. MSN-I showed comparable performance, with a training AUC of 0.720 (SD = 0.127; permutation P = 0.007) and an external validation AUC of 0.804 (95% CI: 0.627–0.952; accuracy = 76.7%, sensitivity = 84.2%, specificity = 63.6%). The external AUC of MSN-II was numerically higher than that of MSN-I, but this difference was not statistically significant (AUC comparison test, P = 0.615; **Supplementary Table 2**). In benchmark comparisons, MSN-II significantly outperformed ALFF, the FreeSurfer-derived subcortical volume benchmark, and baseline HAMD-17 (all P < 0.05; **Supplementary Table 2**). Additionally, in an exploratory ROI-level analysis, MSN-II features showed higher absolute correlations with ΔHAMD-17 than MSN-I features across the 26 limbic ROIs (Wilcoxon paired test, P = 0.005), providing supportive but exploratory evidence for second-order texture integration. The remaining feature sets did not demonstrate significant predictive power. ReHo across all 246 Brainnetome ROIs showed non-significant training performance (AUC = 0.586, SD = 0.116; permutation P = 0.172) with limited generalization to the external validation cohort (AUC = 0.641). ALFF did not reach significance in the training cohort (AUC = 0.538, SD = 0.142; permutation P = 0.329; external AUC = 0.584). Subcortical volumes achieved a training AUC of 0.606 (SD = 0.204; permutation P = 0.118) and an external validation AUC of 0.567. Baseline HAMD-17 yielded a training AUC of 0.541 (SD = 0.104; permutation P = 0.464) and an external validation AUC of 0.548. We additionally tested Brainnetome ROI-wise GMV and FreeSurfer-derived cortical morphometry (mean thickness, hemisphere-wise curvature, and sulcal depth); however, L1 regularization reduced all coefficients to zero for both feature sets, indicating absence of discriminative signal; these feature sets are therefore excluded from **Table 2**.

**Table 2:** Prediction performance across feature sets.

Feature	Training AUC (mean ± SD)	Perm. P	External AUC	95% CI	ACC	SN	SP	PPV	NPV
MSN-II	0.792 ± 0.158	<0.001***	0.856	[0.693, 0.978]	0.833	0.737	1.000	1.000	0.688
MSN-I	0.720 ± 0.127	0.007**	0.804	[0.627, 0.952]	0.767	0.842	0.636	0.800	0.700
ReHo	0.586 ± 0.116	0.172	0.641	[0.428, 0.835]	0.567	0.632	0.455	0.667	0.417
ALFF	0.538 ± 0.142	0.329	0.584	[0.356, 0.801]	0.533	0.579	0.455	0.647	0.385
Subcortical volumes	0.606 ± 0.204	0.118	0.567	[0.358, 0.783]	0.600	0.632	0.545	0.706	0.462
HAMD-17	0.541 ± 0.104	0.464	0.548	[0.321, 0.765]	0.433	0.316	0.636	0.600	0.350

The regularization parameter C was independently optimized for each feature set via inner 5-fold cross-validation. Perm. P = permutation test P-value (2,000 permutations). ***P < 0.001; **P < 0.01. MSN-I and MSN-II were analyzed within the 26 limbic ROIs; ReHo and ALFF were analyzed across all 246 Brainnetome Atlas ROIs; subcortical volumes (6 features) and HAMD-17 (1 feature) are subject-level measures. For GMV and cortical morphometry, L1 regularization reduced all coefficients to zero (null models); classification metrics are therefore not reported. ACC = accuracy; SN, sensitivity; SP, specificity; PPV, positive predictive value; NPV, negative predictive value. Subcortical volumes refer to six FreeSurfer-derived whole-structure subcortical volume measures, including bilateral hippocampus, amygdala, and thalamus. These measures are distinct from Brainnetome ROI-wise GMV. Because subcortical volumes and cortical features originate from different anatomical frameworks, **Table 2** should be interpreted as a benchmark comparison across commonly used predictor families, rather than as a matched anatomical comparison.

**Figure 4:**
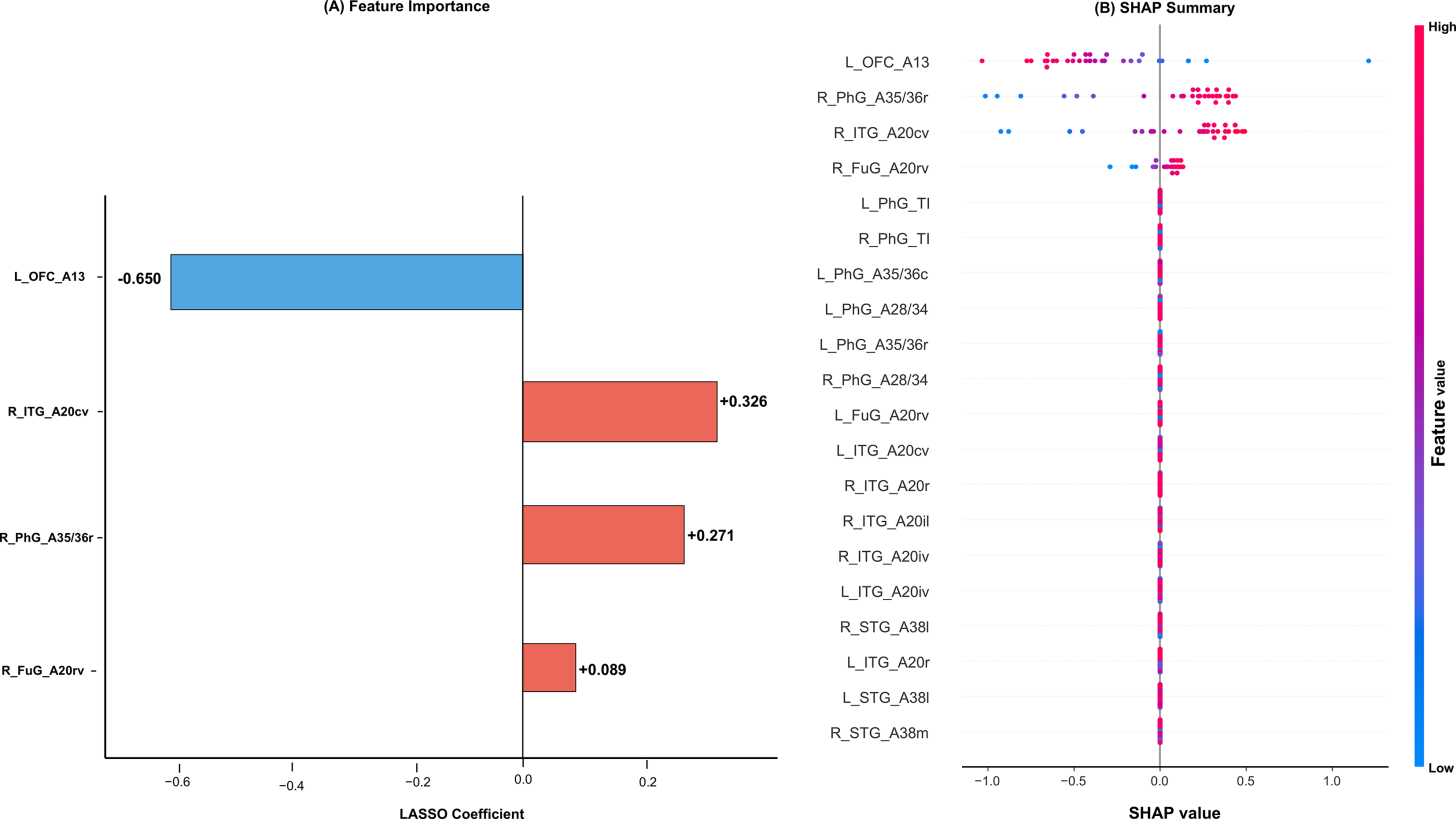
Feature importance. Feature importance of the MSN-II model. **(A)** LASSO coefficients for the four non-zero features (C = 0.3). Blue indicates that lower feature values predict response, whereas red indicates that higher feature values predict response. **(B)** SHAP summary plot for the external validation cohort. In the SHAP summary plot, red indicates higher feature values and blue indicates lower feature values.

LASSO regression with the optimal regularization parameter (C = 0.3), applied to MSN-II nodal strength derived from the GLCM + GLDM + GLRLM texture combination, identified 4 non-zero coefficients among the 26 limbic regions (**Table 3**). The left orbitofrontal cortex area 13 (L_OFC_A13) had the largest absolute coefficient (**β**=−0.649), indicating that lower baseline MSN-II nodal strength in this region was associated with treatment response. Other contributing features included the right inferior temporal gyrus A20cv (**β**=+0.323), right parahippocampal gyrus A35/36r (**β**=+0.271), and right fusiform gyrus A20rv (**β**=+0.091). SHAP analysis confirmed L_OFC_A13 as the most influential feature (mean absolute SHAP = 0.457). The retained non-zero coefficients and SHAP-based feature contributions are shown in **Figure 4**.

**Table 3:** Feature importance ranked by coefficient magnitude.

Rank	Region	Coefficient (β)	SHAP importance
1	L_OFC_A13 (Left orbitofrontal cortex, area 13)	−0.6492	0.4569
2	R_ITG_A20cv (Right inferior temporal gyrus, A20cv)	+0.3230	0.3381
3	R_PhG_A35/36r (Right parahippocampal gyrus, A35/36r)	+0.2713	0.3594
4	R_FuG_A20rv (Right fusiform gyrus, A20rv)	+0.0910	0.0903

Coefficients from L1-regularized logistic regression (C = 0.3) trained on Site A. Negative coefficients indicate that lower MSN-II nodal strength is associated with treatment response; positive coefficients indicate that higher values are associated with response. SHAP, SHapley Additive exPlanations; SHAP importance represents mean absolute SHAP values across the external validation cohort. Abbreviations: OFC, orbitofrontal cortex; PhG, parahippocampal gyrus; ITG, inferior temporal gyrus; FuG, fusiform gyrus.

### 3.3 Baseline differences and longitudinal changes

**Figure 5:**
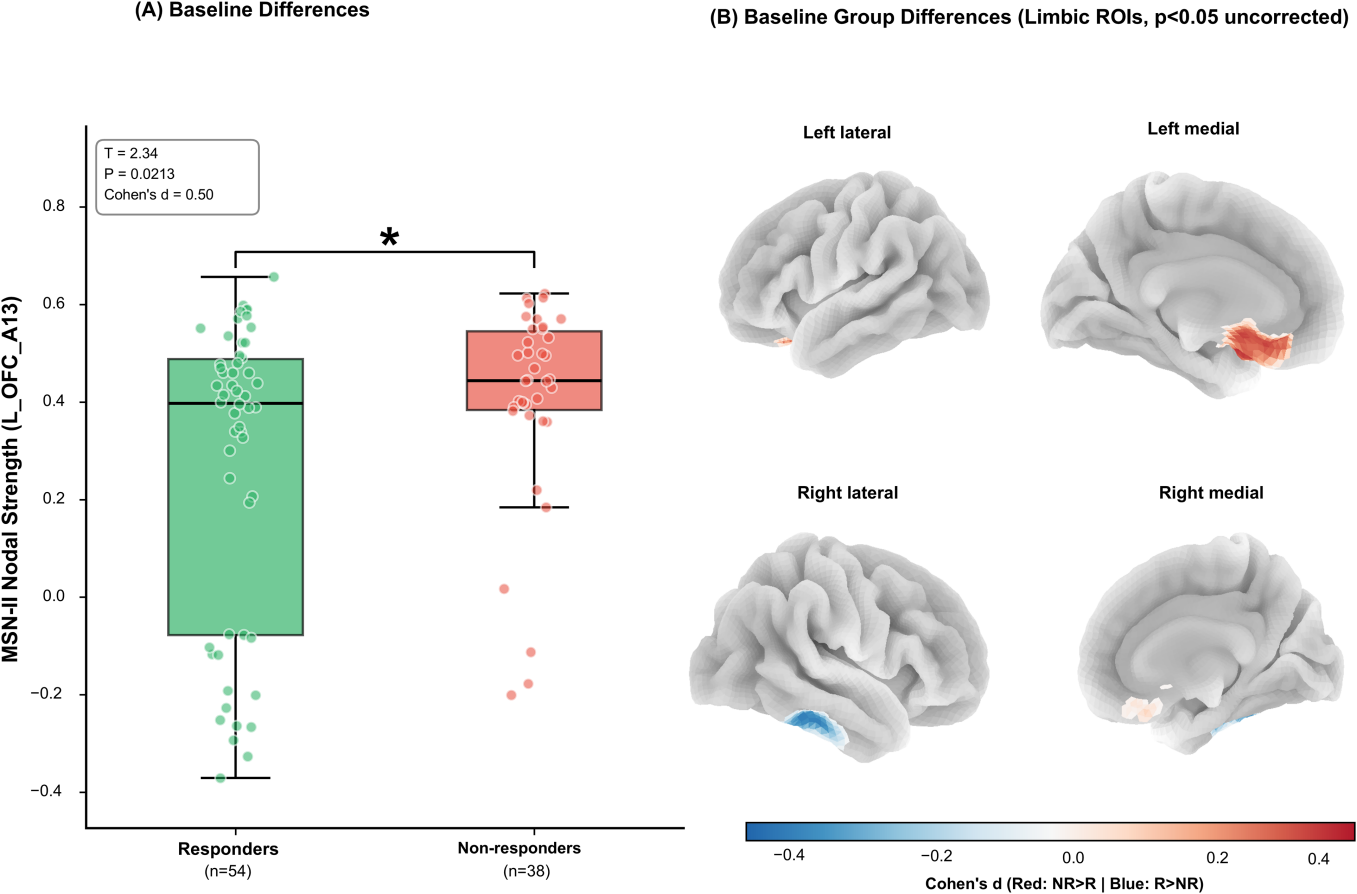
Baseline MSN-II differences by treatment response. Baseline MSN-II differences. **(A)** L_OFC_A13 nodal strength in responders vs. non-responders (P = 0.021, Cohen’s d = 0.50). **(B)** Surface rendering of Cohen’s d across limbic ROIs (uncorrected P < 0.05). MSN-II nodal strength is a unitless network measure.

**Figure 6:**
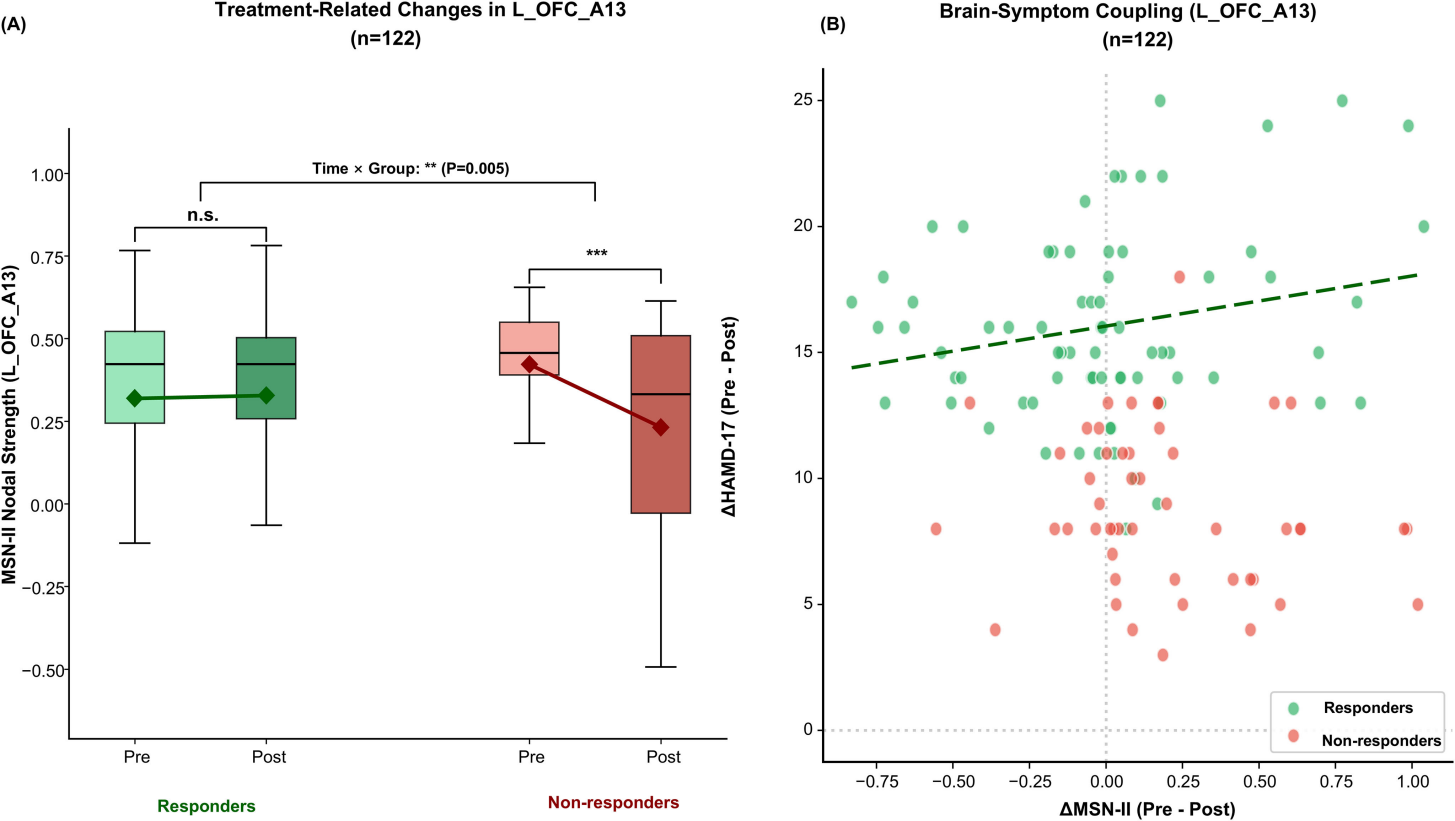
Treatment-related changes and brain–symptom coupling in L_OFC_A13. Treatment-related changes and brain–symptom coupling in L_OFC_A13. **(A)** Non-responders showed significant pre-to-post decreases in MSN-II nodal strength (paired t-test P < 0.001), whereas responders remained stable (P = 0.853). The Time × Group interaction was significant (P = 0.005). **(B)** Brain–symptom coupling between ΔMSN-II and ΔHAMD-17 differed between responders and non-responders (interaction P = 0.047); in responders, the association between ΔMSN-II and symptom reduction showed a trend (P = 0.065). ΔMSN-II was defined as pre-treatment minus post-treatment MSN-II nodal strength. MSN-II nodal strength is a unitless network measure. ΔHAMD-17 was defined as pre-treatment minus post-treatment HAMD-17 score and is expressed in HAMD-17 points.

At baseline, non-responders exhibited significantly higher MSN-II nodal strength in L_OFC_A13 compared with responders (0.404 ± 0.209 vs. 0.268 ± 0.310; P = 0.021; Cohen’s d = 0.50; **Figure 5**). Treatment-related changes in L_OFC_A13 are shown in **Figure 6A**. Non-responders demonstrated a significant decrease from pre- to post-treatment (pre–post difference = +0.191 ± 0.333; paired t-test P < 0.001), whereas responders showed no significant change (pre–post difference = −0.009 ± 0.402; paired t-test P = 0.853). The Time × Group interaction was significant (t = 2.847, P = 0.005), confirming differential treatment-related trajectories between response groups.

### 3.4 Brain-symptom coupling

To formally test whether brain–symptom coupling differed between responders and non-responders, a multiple linear regression model was fitted to the full sample (n = 122) with ΔHAMD-17 as the outcome, including ΔMSN-II in L_OFC_A13, response group (binary), and their interaction term as predictors. The interaction term (ΔMSN-II × Response Group) was significant (**β** = +3.595, SE = 1.793, P = 0.047; **Figure 6B**), indicating that the association between OFC network reorganization and symptom change differed significantly between groups. Specifically, in responders, greater ΔMSN-II in L_OFC_A13 was positively associated with symptom reduction (Pearson R = 0.217, P = 0.065), whereas no such association was observed in non-responders (R = −0.171, P = 0.241).

### 3.5 Exploratory continuous-outcome and correlation analyses

To address whether the retained imaging features also related to continuous clinical improvement, we performed exploratory continuous-outcome prediction analyses of ΔHAMD-17. The four MSN-II features retained by the primary LASSO logistic classifier were entered into ordinary least squares (OLS) linear regression, because the feature set had already been determined by the classification model and the aim was to evaluate their linear explanatory value without a second round of penalized feature selection. In the external validation cohort, baseline HAMD-17 alone predicted ΔHAMD-17 (R² = 0.131, R = 0.409, P = 0.025). Adding the four retained MSN-II features improved prediction (R² = 0.231, R = 0.505, P = 0.0044; MAE = 3.468, RMSE = 4.543). Sensitivity analyses using ridge and LASSO regression for the combined model yielded consistent results (ridge: R = 0.517, P = 0.0035; LASSO: R = 0.492, P = 0.0057; **Supplementary Table 5**).

We also examined Pearson correlations between the retained MSN-II features and clinical outcomes. Baseline MSN-II in R_ITG_A20cv and R_PhG_A35/36r was significantly correlated with ΔHAMD-17 (R = 0.180, P = 0.048; R = 0.231, P = 0.011, respectively), and ΔMSN-II in R_PhG_A35/36r was also correlated with ΔHAMD-17 (R = 0.195, P = 0.031). By contrast, L_OFC_A13 did not show a significant univariate linear correlation with ΔHAMD-17, supporting its role as a multivariate classifier feature rather than a simple linear correlate (**Supplementary Table 3**).

## 4 Discussion

This two-center study demonstrates that MSN features from limbic regions can predict clinical response to taVNS in MDD. The MSN-II model achieved an AUC of 0.856 in external validation, with significant above-chance performance confirmed by permutation testing in the training cohort (P < 0.001). MSN-I showed comparable performance (external AUC = 0.804; training permutation P = 0.007). Although MSN-II showed the highest numerical external AUC, the improvement over MSN-I was not statistically significant. Both MSN representations showed substantially stronger external performance than conventional regional measures, including ReHo, ALFF, FreeSurfer-derived subcortical volumes, and baseline HAMD-17 (**Table 2**). The left orbitofrontal cortex emerged as the key predictive region, with baseline differences and differential longitudinal trajectories distinguishing responders from non-responders.

MSN-II quantifies higher-order structural organization by integrating connectivity profiles across multiple radiomics texture groups. By concatenating row-standardized off-diagonal features from GLCM, GLDM, and GLRLM similarity matrices and correlating these composite profiles, MSN-II captures inter-regional similarity in multi-scale texture patterns, representing a data-driven hierarchical characterization of brain morphometric organization.

In the present study, MSN-II showed a numerically higher external validation AUC than MSN-I (0.856 vs. 0.804), but this difference was not statistically significant. Therefore, the present data should not be interpreted as definitive evidence that MSN-II is superior to MSN-I. Instead, the findings suggest that second-order texture integration may provide complementary and potentially useful information, an interpretation further supported by exploratory ROI-level correlations but requiring confirmation in larger cohorts. Importantly, both MSN-I and MSN-II outperformed conventional morphometric and resting-state functional measures, indicating that network-level characterization of brain morphology carries predictive information not available from regional properties alone.

Among conventional neuroimaging measures, ReHo across all 246 Brainnetome ROIs showed non-significant training performance (permutation P = 0.172) with limited generalization to the external validation cohort (AUC = 0.641), suggesting possible overfitting of regional functional homogeneity features. ALFF did not reach significance in the training cohort (permutation P = 0.329), and subcortical volumes showed a non-significant effect (external AUC = 0.567, permutation P = 0.118). This pattern reinforces the advantage of network-based over region-based approaches for cross-site treatment prediction.

The orbitofrontal cortex has been consistently identified as a key region in MDD pathophysiology. Large-scale ENIGMA analyses have reported cortical abnormalities in depression, and more recent work has highlighted dissociable structural and functional alterations of medial and lateral OFC subregions in MDD (8, 17). Functional studies have demonstrated that the OFC plays a central role in reward processing and emotion regulation, and that these functions are disrupted in MDD, contributing to anhedonia and negative affective bias (16). The OFC is also critically involved in flexible decision-making through its dense connections with the amygdala, hippocampus, and medial prefrontal cortex (15). In the context of vagus nerve stimulation, vagal afferents project to the nucleus tractus solitarius (NTS) and subsequently engage downstream brainstem neuromodulatory nuclei and distributed thalamo-cortical and limbic-prefrontal pathways that include the OFC (30, 31). Our findings extend this literature by suggesting that the higher-order structural organization of the OFC may influence the capacity for beneficial neuromodulatory effects of taVNS. Specifically, non-responders exhibited significantly higher baseline MSN-II nodal strength in L_OFC_A13, suggesting that atypical higher-order structural covariance in this region may be associated with reduced treatment responsiveness. In addition, non-responders showed a significant decrease in L_OFC_A13 MSN-II after treatment, whereas responders showed no significant change, indicating distinct longitudinal trajectories. Finally, a significant interaction between ΔMSN-II and response group (β = +3.595, P = 0.047) indicated that the relationship between OFC network reorganization and symptom change differed significantly across groups. In responders, greater ΔMSN-II was positively associated with symptom reduction, further supporting a meaningful link between L_OFC_A13 network organization and clinical benefit from taVNS. The elevated baseline MSN-II in non-responders could signal rigid structural covariance that resists neuromodulatory change. Their post-treatment decrease, occurring without symptom relief, may represent non-therapeutic reorganization rather than beneficial plasticity. In contrast, the relative stability of OFC network organization in responders, together with significant symptom reduction and differential brain–symptom coupling, is consistent with the view that baseline circuit architecture can constrain treatment response trajectories (13). Importantly, the right homotopic OFC region R_OFC_A13 was included in the *a priori* limbic mask but was not retained by the LASSO model. Supplementary analysis showed no significant association between R_OFC_A13 ΔMSN-II and ΔHAMD-17 (R = 0.003, P = 0.970), suggesting that the L_OFC_A13 finding was not caused by asymmetric ROI selection. Nevertheless, the lateralized nature of this finding should be interpreted cautiously until replicated in larger datasets.

Beyond the OFC, the other retained regions may also be relevant to taVNS response. The right inferior temporal and fusiform regions are high-level association cortices involved in perceptual, affective, and semantic integration, and their MSN-II organization may index broader limbic-temporal network integrity. The parahippocampal gyrus is closely related to contextual memory processing, stress-related affective regulation, and hippocampal network plasticity. Because taVNS engages vagal afferent pathways that project through brainstem nuclei to distributed limbic-prefrontal circuits, the higher-order structural organization of these orbitofrontal, temporal, fusiform, and parahippocampal regions may reflect individual differences in the capacity for neuromodulatory change.

Several limitations should be considered. First, the modest sample size of the validation cohort (n = 30) limits the precision of external performance estimates, as reflected in the wide 95% confidence interval (0.693–0.978). Therefore, the current findings should be interpreted as demonstrating promising cross-site generalizability rather than definitive robust prediction. Second, baseline SDS scores differed slightly between sites (P = 0.047). Given that SDS is a self-report scale, this difference may partly reflect subjective reporting variability, while clinician-rated HAMD-17 and HAMA scores were comparable between sites. Third, the binary response definition, although stringent, may not capture the full spectrum of clinical improvement. Fourth, the 1-month antidepressant washout period may have been insufficient for medications with long half-lives. Fifth, although MSN-II showed a numerically higher external AUC than MSN-I (0.856 vs. 0.804), the advantage was modest and not statistically significant; the specific methodological benefit of second-order analysis therefore warrants confirmation in larger samples. Sixth, ReHo showed non-significant training performance (permutation P = 0.172) and limited generalization to the external cohort (AUC = 0.641), while ALFF did not reach training significance (permutation P = 0.329), suggesting that whole-brain resting-state functional measures may require larger samples or more targeted analytical approaches to achieve reliable cross-site prediction. Although we adopted a stringent response threshold (≥60% HAMD-17 reduction) to mitigate label noise and phenotypic heterogeneity in predictive modeling (19–21), future multi-center studies with larger samples, sham control groups, and nested cross-validation frameworks are needed to confirm the robustness of these findings.

## 5 Conclusions

In conclusion, this study demonstrated that morphometric similarity network features derived from baseline structural MRI, including MSN-II, provide promising cross-site prediction of taVNS response in MDD (external AUC = 0.856; training permutation P < 0.001). The orbitofrontal cortex, particularly L_OFC_A13, emerged as a central biomarker, with baseline differences and differential treatment-related changes distinguishing responders from non-responders. These findings support MSN as a promising neuroimaging framework for individualized treatment planning, while requiring validation in larger independent cohorts.

## Data Availability

The raw data supporting the conclusions of this article will be made available by the authors, without undue reservation.
