# Supplementary material for: Second-order morphometric similarity networks predict response to transcutaneous auricular vagus nerve stimulation in major depressive disorder: a two-center study
Source: Front Psychiatry. 2026 Jul 8;17:1858627. doi: 10.3389/fpsyt.2026.1858627 (PMC13388863; doi:10.3389/fpsyt.2026.1858627)
Supplement: Supplementary file 1 [file Table1.docx]

# **Supplementary Materials**

**Supplementary Table S1. List of 26 Limbic ROIs**

| **Label ID** | **Abbreviation** | **Description** |
| --- | --- | --- |
| 27 | L_MFG_A10l | Left middle frontal gyrus, A10l |
| 45 | L_OFC_A11l | Left orbital gyrus, A11l |
| 47 | L_OFC_A11m | Left orbital gyrus, A11m |
| 48 | R_OFC_A11m | Right orbital gyrus, A11m |
| 49 | L_OFC_A13 | Left orbital gyrus, A13 |
| 50 | R_OFC_A13 | Right orbital gyrus, A13 |
| 69 | L_STG_A38m | Left superior temporal gyrus, A38m |
| 70 | R_STG_A38m | Right superior temporal gyrus, A38m |
| 77 | L_STG_A38l | Left superior temporal gyrus, A38l |
| 78 | R_STG_A38l | Right superior temporal gyrus, A38l |
| 89 | L_ITG_A20iv | Left inferior temporal gyrus, A20iv |
| 90 | R_ITG_A20iv | Right inferior temporal gyrus, A20iv |
| 93 | L_ITG_A20r | Left inferior temporal gyrus, A20r |
| 94 | R_ITG_A20r | Right inferior temporal gyrus, A20r |
| 96 | R_ITG_A20il | Right inferior temporal gyrus, A20il |
| 101 | L_ITG_A20cv | Left inferior temporal gyrus, A20cv |
| 102 | R_ITG_A20cv | Right inferior temporal gyrus, A20cv |
| 103 | L_FuG_A20rv | Left fusiform gyrus, A20rv |
| 104 | R_FuG_A20rv | Right fusiform gyrus, A20rv |
| 109 | L_PhG_A35/36r | Left parahippocampal gyrus, A35/36r |
| 110 | R_PhG_A35/36r | Right parahippocampal gyrus, A35/36r |
| 111 | L_PhG_A35/36c | Left parahippocampal gyrus, A35/36c |
| 115 | L_PhG_A28/34 | Left parahippocampal gyrus, A28/34 |
| 116 | R_PhG_A28/34 | Right parahippocampal gyrus, A28/34 |
| 117 | L_PhG_TI | Left parahippocampal gyrus, temporal isthmus |
| 118 | R_PhG_TI | Right parahippocampal gyrus, temporal isthmus |

**Supplementary Table S2. AUC Comparison Tests in the External Validation Cohort**

| **Comparison** | **AUC1** | **AUC2** | **P** |
| --- | --- | --- | --- |
| MSN-II vs. MSN-I | 0.856 | 0.804 | 0.615 |
| MSN-II vs. ReHo | 0.856 | 0.641 | 0.132 |
| MSN-II vs. ALFF | 0.856 | 0.584 | 0.009 |
| MSN-II vs. Subcortical volumes | 0.856 | 0.567 | 0.014 |
| MSN-II vs. HAMD-17 | 0.856 | 0.548 | 0.033 |
| MSN-II vs. RSFC limbic strength | 0.856 | 0.722 | 0.308 |

*Note: AUCs are the external validation values reported in Table 2. Subcortical volumes are six FreeSurfer-derived whole-structure subcortical volume measures (bilateral hippocampus, amygdala, and thalamus), distinct from Brainnetome ROI-wise GMV.*

**Supplementary Table S3. Correlations Between Retained MSN-II Features and Clinical Outcomes**

| **Region** | **β** | **SHAP** | **R (baseline vs. HAMDpre)** | **P** | **r (baseline vs. ΔHAMD)** | **P** | **R (ΔMSN vs. ΔHAMD)** | **P** |
| --- | --- | --- | --- | --- | --- | --- | --- | --- |
| L_OFC_A13 | -0.649 | 0.457 | +0.046 | 0.615 | -0.093 | 0.308 | -0.116 | 0.203 |
| R_ITG_A20cv | +0.323 | 0.338 | -0.020 | 0.827 | +0.180 | 0.048 | +0.165 | 0.069 |
| R_PhG_A35/36r | +0.271 | 0.359 | +0.030 | 0.741 | +0.231 | 0.011 | +0.195 | 0.031 |
| R_FuG_A20rv | +0.091 | 0.090 | -0.009 | 0.920 | +0.150 | 0.100 | +0.104 | 0.254 |

*Note: β denotes the LASSO coefficient from the primary classification model. ΔMSN = pre-treatment minus post-treatment MSN-II nodal strength; ΔHAMD = HAMD-17 pre-treatment minus post-treatment. Correlations were exploratory Pearson correlations.*

**Supplementary Table S4. Supplementary Analysis of Left and Right OFC**

| **Region** | **Included in limbic mask** | **Selected by LASSO** | **ΔMSN-II vs. ΔHAMD-17** |
| --- | --- | --- | --- |
| L_OFC_A13 | Yes | Yes | r = -0.116, P = 0.203 |
| R_OFC_A13 | Yes | No | r = +0.003, P = 0.970 |

**Supplementary Table S5. Exploratory Continuous-Outcome Prediction of ΔHAMD-17**

| **Model** | **External R²** | **External R** | **P** | **MAE** | **RMSE** |
| --- | --- | --- | --- | --- | --- |
| HAMD-17 baseline only (OLS) | 0.131 | 0.409 | 0.025 | 3.924 | 4.828 |
| MSN-II selected 4 (OLS) | 0.103 | 0.390 | 0.033 | 4.093 | 4.905 |
| MSN-II selected 4 + HAMD-17 (OLS) | 0.231 | 0.505 | 0.0044 | 3.468 | 4.543 |

*Note: The four MSN-II features were pre-selected by the primary LASSO logistic classifier. OLS was used as the primary continuous-outcome analysis because the feature set had already been defined by the primary classification model. Sensitivity analyses using ridge and LASSO for the combined model yielded consistent results (ridge: r = 0.517, P = 0.0035; LASSO: r = 0.492, P = 0.0057). LASSO applied to imaging features alone produced a near-null model and was treated as sensitivity only.*
